# Antigout Effects of* Plantago asiatica*: Xanthine Oxidase Inhibitory Activities Assessed by Electrochemical Biosensing Method

**DOI:** 10.1155/2018/1364617

**Published:** 2018-02-22

**Authors:** Jin-Xiang Zeng, Juan Wang, Shou-Wen Zhang, Ji-Xiao Zhu, Min Li, Wei-Hua Huang, Jin-Yi Wan, Hai-Qiang Yao, Chong-Zhi Wang, Chun-Su Yuan

**Affiliations:** ^1^The Research Center of Chinese Medicine Resource and National Medicine of Jiangxi University of Traditional Chinese Medicine, No. 818, Xingwan Road, Nanchang 330004, China; ^2^Institute of Clinical Pharmacology, Hunan Key Laboratory of Harmacogenetics, Central South University, Changsha 410078, China; ^3^School of Pharmacy, Jiangsu University, 301 Xuefu Road, Zhenjiang 212013, China; ^4^Beijing University of Chinese Medicine, No. 11, North 3rd Ring Road, Beijing 100029, China; ^5^Tang Center for Herbal Medicine Research and Department of Anesthesia and Critical Care, University of Chicago, 5841 South Maryland Avenue, MC 4028, Chicago, IL 60637, USA

## Abstract

The XOD inhibitory effects of Plantaginis Semen, that is, the seeds of* P. asiatisca*, and its representative four single compounds, acteoside, 1H-indolo-3-carbaldehyde, isoacteoside, and myristic acid, were evaluated by electron transfer signal blocking activities (ETSBA), which is based on the electron transfer signal of XOD enzymatic reaction. The blocking activities were detected using an electrochemical biosensing method. Compared with control, significant effects were observed after the addition of* P. asiatica *extract, acteoside, and 1H-indolo-3-carbaldehyde (all *p* < 0.05). The IC50 values of the extract and acteoside are 89.14 and 7.55 *μ*g·mL^−1^, respectively. The IC20 values of the extract, acteoside, and 1H-indolo-3-carbaldehyde are 24.28, 3.88, and 16.16 *μ*g·mL^−1^, respectively. Due to the relatively lower inhibitory potential of 1H-indolo-3-carbaldehyde, its IC50 was not obtained. In addition, isoacteoside and myristic acid did not show any XOD inhibitory effects. Our data demonstrated that the XOD inhibitory effects of the extract, acteoside, and 1H-indolo-3-carbaldehyde can be accurately evaluated by the ETSBA method. The results from this study indicated that Plantaginis Semen significantly inhibited XOD activities to reduce hyperuricemia and treat gout. The study also proves that measuring the electron transfer signal blocking activities is a simple, sensitive, and accurate method to evaluate the XOD inhibitory effects.

## 1. Introduction

Gout is medical condition caused by long-term hyperuricemia and has become increasingly common over the last 15 years. This disease can be a severely disabling disorder inducing significant pain and limiting daily activity and social functioning, leading to a poor health-related quality of life [1]. The main method for treating gout and hyperuricemia is to lower serum uric acid levels [2]. Xanthine oxidase (XOD) inhibitors of allopurinol and febuxostat have been commonly used medications to decrease the circulating uric acid levels [3, 4]. However, the use of these drugs is associated with various side effects such as skin rashes, systemic vasculitis, and even renal failure [1, 2]. Thus, effective XOD inhibitors with reduced side effects are urgently needed for the treatment of this troublesome medical disorder.

Recently, XOD inhibitors from natural products have attracted a great deal of attention due to their nontoxic properties [5–7]. For this reason, it is critically important to accurately evaluate the XOD inhibitory effects of natural products.

**Figure 1:**
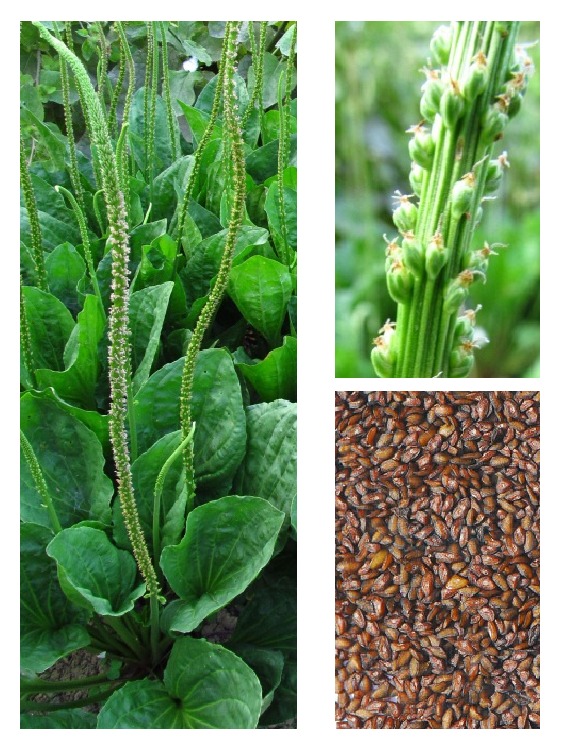
Morphology of* P. asiatica*. The scapes and seeds are shown in the upper right and lower right of the figure.

*Plantago asiatica* L. is a perennial herbal plant frequently used as an ingredient in dietary supplements, and the dried ripe seeds of* P. asiatica*, that is, Plantaginis Semen, are one of the most popular folk herbal medicines in China and other Asian countries as plant-based diuretics (Figure 1). It is reported that this botanical possesses XOD inhibitory activity* in vitro* and* in vivo* and thus can be used for the development of a safer and more effective XOD inhibitor against hyperuricemia [5, 8].

It has been shown that XOD inhibitors not only affect the reacting products, but also block the electron transfer activities of the enzymatic reactions [9–11]. By quantifying enzymatic reaction products, the XOD inhibitory effects can be evaluated using spectrophotometric [12, 13], HPLC [14, 15], or the electrochemical biosensing analyses [16]. Since the constituents in* P. asiatica*, like most other botanicals, are very complex, the aforementioned conventional methods have limitations. For example, ultraviolet visible (UV) spectrophotometric method may easily give false results as certain compounds in a given botanical can absorb light [12, 13], while HPLC-UV/MS requires complicated and time consuming pretreatment procedures and expensive apparatus [14, 15].

The electrochemical biosensing method has been shown to have a number of advantages such as simplicity, rapidity, high sensitivity, and low costs. An electrochemical biosensing method was used to evaluate the XOD inhibitory effect, by quantifying enzymatic reaction product, hydrogen peroxide, detected at a single potential using chronoamperometry [16]. However, the detection may be disturbed by the electrochemical activities of the tested compounds. In our recent study, an electrochemical biosensing technique was adopted to investigate the XOD inhibition of single compounds by electron transfer signal blocking activities (ETSBA), which possessed many advantages compared with other traditional methods [17]. However, whether ETSBA can be utilized for the evaluation of the XOD effects of a botanical extract containing complex constituents has not been previously investigated.

In relation to XOD activity, the compounds in natural products could be classified into two groups: with or without the XOD inhibition effect. Each group could be further classified into two subgroups: with or without electrochemical activity. In this work, the XOD inhibitory activity of* Plantaginis Semen* extract and its four single compounds of acteoside, 1H-indole-3-carbaldehyde, isoacteoside, and myristic acid, which represent those four groups, was evaluated using the ETSBA. We observed that the extract has very significant inhibitory effects and that the variable activities of the four tested single compounds can also be accurately measured by this simple ETSBA testing. Our data supported the notion that the ETSBA successfully provided a simple and accurate evaluation of the XOD inhibition activities of natural products with their potential inhibition properties.

## 2. Experimental

### 2.1. Chemicals

**Figure 2:**
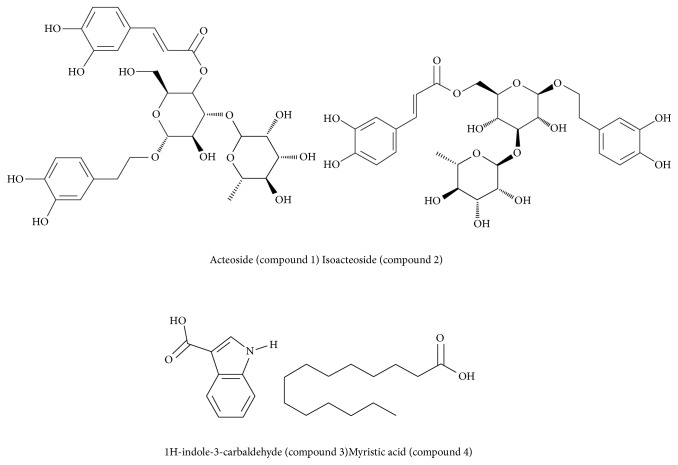
The chemical structures of four representative compounds identified in Plantaginis Semen, that is, acteoside, isoacteoside, 1H-indole-3-carbaldehyde, and myristic acid.

Plantaginis Semen was purchased from a cultivation base in Jiujiang city and identified by Professor Shouwen Zhang of Jiangxi University of Traditional Chinese Medicine. Xanthine oxidase (XOD) (EC1.17.3.2, 12.1 U/mg) was obtained from the Nanjing Jiancheng Bioengineering Institute (Nanjing, China). Shorted double-wall carbon nanotubes (DCNTs) with 95% purity were purchased from Shenzhen Nanotech Port Co. (Shenzhen, China) and purified by refluxing the as-received DCNTs in 2.6 M HNO_3_ for 10 h [18]. Xanthine and allopurinol were purchased from Sigma (St. Louis, MO, USA). All reagents were of analytical grade and were used as received without further purification. Phosphate buffer solution (PBS, 1/15 M) was used as a supporting electrolyte in the measurements and double distilled water was used throughout. Representative compounds of acteoside, isoacteoside, 1H-indole-3-carbaldehyde, and myristic acid were isolated from Plantaginis Semen*; *the purity was found to be >95% [19, 20] and their chemical structures are shown in Figure 2.

### 2.2. Apparatus

Cyclic voltammetry measurements were performed with an Autolab Potentiostat/Galvanostat electrochemical station (Eco Chemie, Netherlands). A conventional three-electrode system with the modified glassy carbon electrode (GCE, 3 mm in diameter) as the working electrode, an Ag∣AgCl∣KCl (AE, 3 mol/L) reference electrode, and a platinum wire electrode as the counter electrode (all from CH Instruments, China) were used. All applied potentials were measured and reported versus AE and all experiments were carried out at room temperature. Scanning electron microscopy (SEM) images were obtained by using a Nova NanoSEM450 field emission SEM (FEI, USA).

### 2.3. Preparation of DCNTs and DCNTs/XOD Modified GCE

The GCEs were carefully polished with emery paper and aqueous slurries of fine alumina powders (0.3 and 0.05 *μ*m) on a polishing cloth until a mirror finish was obtained. After 10 min of sonication, the electrodes were transferred to the electrochemical cell for cleaning by cyclic voltammetry between −0.5 V and +1.2 V at 50 mV/s in PBS (1/15 M, pH 7.2), until a stable profile was obtained. The prepared electrodes were dried under a high purity nitrogen stream and used for modification immediately. 1.0 mg DCNTs was dispersed in 1.0 mL doubly distilled water with the aid of sonication. The DCNTs modified electrode was prepared by casting 3 *μ*L of the dispersion on the surface of a GCE and air-dried at room temperature. The DCNTs modified GCE was further casted by 6 *μ*L 0.147 mM XOD and air-dried at room temperature. The DCNTs and DCNTs/XOD modified electrodes were denoted as DCNTs/GCE and XOD/DCNTs/GCE hereafter, respectively. When not in use, the modified electrodes were stored in a 4°C refrigerator.

### 2.4. Preparation of the Extract of* Plantaginis Semen*

5 g of* Plantaginis Semen *was crushed and extracted with 65% ethanol by reflux for 2 h and repeated 3 times. The solutions were combined and filtered, and the solvent was evaporated under vacuum. 0.926 g deposit was obtained, and the resulting deposit rate is calculated to be 18.5%. The resulting deposits were redispersed in 10 mL water and filtered and then used directly for experiments.

### 2.5. Evaluation Procedures of Electron Transfer Signal Blocking Activities

The PBS solutions were purged with highly purified nitrogen for at least 15 min prior to the series of experiments. All measurements were performed under a nitrogen atmosphere. The XOD/DCNTs/GCE was immersed in PBS and the inhibition reaction was initiated by adding an appropriate concentration of extract or compound to the PBS for about 10 min. Then, xanthine was added. Five minutes later, cyclic voltammetry was performed immediately at the potential range from −600 to 800 mV with a scan rate of 100 mV/s. The response current of the electron transfer signal at about 610 mV was marked with the change value between the peak current and the background current. The details for calculating the response current are listed as follows.

At first, the response current of xanthine in the absence of inhibitors was calculated by two steps measurements. Firstly, the electrode was immersed into the PBS solution without addition of xanthine and inhibitors, and then the current located at 610 mV was recorded. This current is the background current 1. Secondly, xanthine was added to the solution standing for 5 minutes; the peak current of xanthine located at 610 mV was recorded and noted as current 2. Hence, current 2 is subtracted from background current 1; the obtained result is the response current of xanthine (Δ*Ip*_0_).

Then, the response current of xanthine in the presence of inhibitor was measured via the following steps. Firstly, the inhibitor was added to the PBS solution standing for 10 minutes, and then xanthine was added to the solution standing for further 5 minutes; the peak current located at 610 mV is recorded and noted as current 3, which includes the response current of xanthine and inhibitors. Current 3 is subtracted from background current 1; the obtained result is the response current of xanthine and inhibitors. Here it is noted as current 4. Then, the current of the inhibitor at 610 mV was recorded when there is no xanthine under the same conditions and noted as current 5. Current 5 is subtracted from background current 1; the obtained result is the response current of inhibitors and noted as current 6. Hence, current 4 is subtracted from current 6; the obtained result is the response current of xanthine in the presence of inhibitors (Δ*Ip*_1_).

The obtained Δ*Ip*_0_ and Δ*Ip*_1_ values were used to calculate the inhibition rate of the inhibitors according to the equation

(1) $$\begin{array}{c} IC=\frac{\left[\Delta Ip_{0}-\Delta Ip_{1}\right]}{\Delta Ip_{0}}\times 100\%, \end{array}$$

where IC means the inhibition rate of the corresponding concentration of the sample; Δ*Ip*_0_ and Δ*Ip*_1_ represent the response currents of the xanthine without or with inhibitor, respectively. In this study, IC20 and IC50, which mean the corresponding concentrations of the inhibition rate of 20% and 50%, were used to express the XOD inhibitory effects.

### 2.6. Statistical Analysis

Data were expressed as mean ± standard deviation (*n* = 3) using Sigmaplot 10.0. Significant differences among means of samples were evaluated using a one-way analysis of variance (ANOVA). Blank samples without the addition of XOD inhibitors were used as the control.

## 3. Results and Discussions

### 3.1. SEM Characterization of the Modified GCE

Since carbon nanotubes help the XOD molecule pass its electrons into an electrode in order to produce sensitive current signals [10], the double-wall carbon nanotubes (DCNTs) were adopted to do the electron transfer between GCE and XOD.

**Figure 3:**
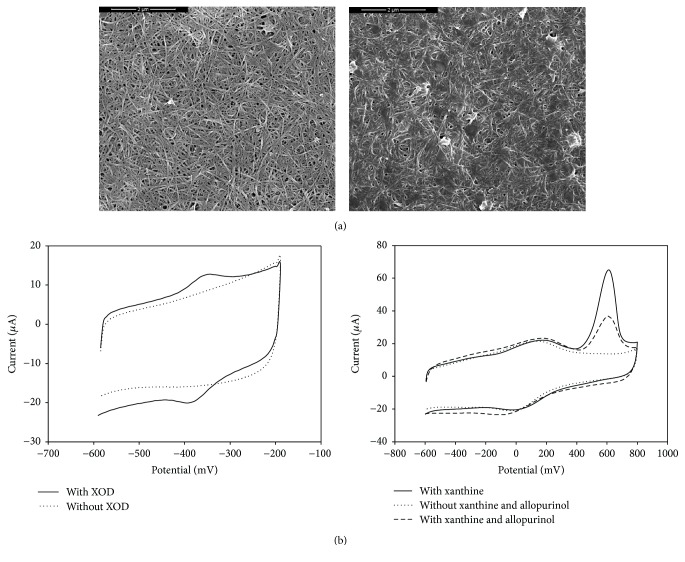
Illustrations of the electron transfer signal blocking activities. (a) Scanning electron microscopy of double-wall carbon nanotubes (DCNTs) modified glassy carbon electrode (DCNTs/GCE, left) and DCNTs plus XOD modified glassy carbon electrode (XOD/DCNTs/GCE, right). (b) Cyclic voltammograms of DCNTs/GCE and XOD/DCNTs/GCE (left), and XOD/DCNTs/GCE in the absence and presence of xanthine, in the presence of xanthine and allopurinol (right). The scan rate of the cyclic voltammograms is 100 mV/s.

Figure 3(a) displays the typical morphology of the DCNTs (left) and XOD/DCNTs (right) characterized by SEM. From Figure 3(a) (left), it can be seen that DCNTs were in the form of small bundles and single nanotubes and distributed homogeneously on the surface of the GCE, exhibiting a special three-dimensional structure. The XOD casted onto the surface of DCNTs (right) did not change the morphology of DCNTs except that the brightness was slightly weakened, which may have been caused by XOD. Such small bundles and single nanotubes homogeneously distributed on GCE and twisted with XOD were expected to be very attractive for detecting electron transfer signal from the enzymatic and electrochemical reaction, yielding a high signal-to-noise ratio [18] for the evaluation of the XOD inhibitory effect of natural products.

### 3.2. Cyclic Voltammetry Characterization of Electron Transfer Signal Blocking Activities

Uric acid and hydrogen peroxide of XOD enzymatic reaction products are widely used to evaluate the inhibitory effect of XOD inhibitors [12–16], but using the electron transfer signal blocking activities (ETSBA) to evaluate XOD inhibitory effects are rarely investigated [17]. Here, the ETSBA was characterized by cyclic voltammetry (CV), and the results are shown in Figure 3(b). It can be seen that there are a couple of stable and well-defined redox peaks of the FAD/FADH_2_ cofactor at the XOD/DCNTs/GCE in PBS (1/15 M, pH 5.3), which are attributed to the excellent electron transfer ability of DCNTs. The anodic peak potential (Epa) and cathodic peak potential (Epc) are located at −350 and −375 mV, respectively. The peak-to-peak potential separation is about 25 mV. The redox peak couple is attributed to the conversion of the oxidized FAD cofactor into a reduced FADH_2_ cofactor of XOD, which is consistent with the literature [10, 11]. Figure 3(b) (right) shows the CVs of XOD/DCNTs/GCE in the absence of and in the presence of 28 *μ*g·mL^−1^ xanthine, or further added with 1.8 *μ*g·mL^−1^ allopurinol. A sharp and sensitive peak current can be seen at about 610 mV. The current of electron transfer signal is confirmed to have exclusively resulted from the over-potential-oxidation of the FAD/FADH_2_ redox couple [9, 21], which is caused by the excellent electron transfer ability and the signal amplifies function of carbon nanotubes. However, with the addition of 1.8 *μ*g·mL^−1^ allopurinol, the peak current decreased sharply, which proved that electron transfer signal blocking activities are affected by allopurinol with high sensitivity. Hence, ETSBA could be undoubtedly applied to evaluate the XOD inhibitory effect of natural products, which has been applied successfully in our recent work [17].

### 3.3. Stability and Reproducibility of Electron Transfer Signal Blocking Activities

Stability and reproducibility of the ETSBA of the XOD/DCNTs/GCE stored in the 4°C refrigerator were also investigated using CVs in PBS (1/15 M, pH 5.3). The response currents of the XOD/DCNTs modified electrodes almost did not change in 24 hours. The relative standard deviation (RSD) of 10 measurements of 28 *μ*g·mL^−1^ xanthine with the same XOD/DCNTs/GCE and 5 different XOD/DCNTs modified GCEs was 2.8% and 3.2%, respectively. These results suggested that there is excellent reproducibility and long-time stability for ETSBA, which is very facilitative for the evaluation of the XOD inhibitory effect.

### 3.4. Evaluate XOD Inhibitory Effect of the* Plantaginis Semen* Extract and Compounds via Electron Transfer Signal Blocking Activities

The reported electrochemical biosensing method for screening XOD inhibitors is based on the enzymatic reaction products of hydrogen peroxide detected at a single potential using chronoamperometry, which may be easily disturbed by electrochemical active constituents [16]. ETSBA in this study is based on the electron transfer signal of the enzymatic reaction and is completely different from that of the reported electrochemical biosensing method, which may further improve the evaluating characteristics. In this study, four compounds with or without XOD inhibitory effects and/or electrochemical activities of acteoside, 1H-indole-3-carbaldehyde, isoacteoside, and myristic acid identified from* Plantaginis Semen *[19, 20] were selected to study ETSBA for the evaluation of XOD inhibitory effects. Furthermore, isomers and compounds with high, low, and no XOD inhibitory effects were also considered. Hence, these selected compounds can represent all the types of the natural compounds. The XOD inhibitory effects and/or electrochemical activities of the extracts and four compounds are described as follows and summarized in Table 1.

#### 3.4.1. Evaluate XOD Inhibitory Effect of Extract via Electron Transfer Signal Blocking Activities

It is very important to accurately evaluate the XOD inhibition effect of extract, as it is the prerequisite and basis for further active constituent research. However, the traditional ultraviolet and visible spectrophotometry (UV) method may give false results easily because the constituents in the extract are very complicated, while HPLC-UV/MS methods need complicated pretreatment procedures and expensive apparatus [12–15], which may significantly hinder the development of new safer and effective XOD inhibitors.

**Figure 4:**
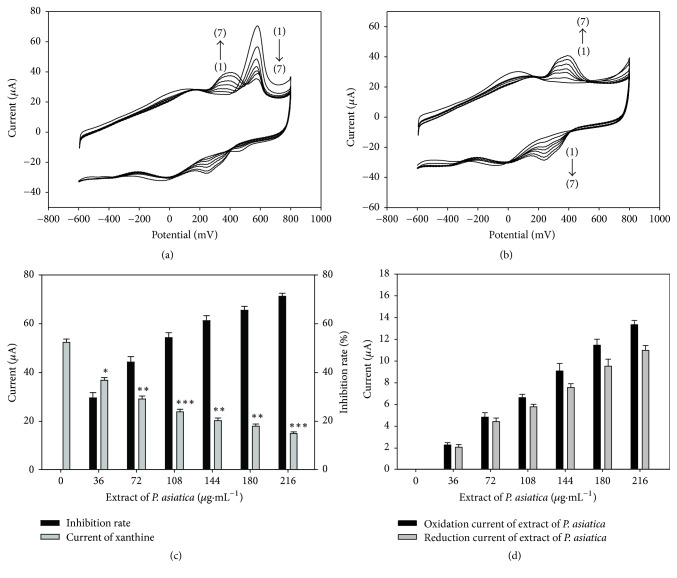
Effects of the representative electron transfer signal blocking activities of Plantaginis Semen on XOD/DCNTs/GCE. (a) In the presence of xanthine with different concentrations of the Plantaginis Semen extract. The lines (1) to (7) are XOD/DCNTs/GCE in the presence of 28 *μ*g·mL^−1^ xanthine and 0, 36, 72, 108, 144, 180, and 216 *μ*g·mL^−1^ of the extract, respectively. (b) In the absence of xanthine with different concentrations of the extract. The lines (1) to (7) are XOD/DCNTs/GCE in the presence of 0, 36, 72, 108, 144, 180, and 216 *μ*g·mL^−1^ of the extract, respectively. (c) Relationship between the inhibition rate and Plantaginis Semen extract concentration. (d) Relationship between the oxidation and reduction current and Plantaginis Semen extract concentration. ^*∗*^*p* < 0.05, ^*∗∗*^*p* < 0.01, and ^*∗∗∗*^*p* < 0.001, compared with control.

It can be seen from Figures 4(a) and 4(b) that there was a couple of redox peaks located in the range from 200 to 500 mV for* Plantaginis Semen *extract, which is caused by the reduction and oxidation of the electrochemical active constituents. The peak currents increased (Figures 4(b) and 4(d)) with the successive addition of 0, 36, 72, 108, 144, 180, and 216 *μ*g·mL^−1^ extract. In this figure, lines (1) to (7) represent the concentration of extract being 0, 36, 72, 108, 144, 180, and 216 *μ*g·mL^−1^, respectively. The peak current of xanthine located at about 610 mV was accordingly decreased with addition of extract. The corresponding current was 52.36 ± 1.35 *μ*A, 35.84 ± 1.12 *μ*A (*p* < 0.05), 27.17 ± 1.18 *μ*A (*p* < 0.01), 22.12 ± 1.05 *μ*A (*p* < 0.001), 19.89 ± 1.08 *μ*A (*p* < 0.01), 18.06 ± 0.84 *μ*A (*p* < 0.01), and 14.04 ± 0.65 *μ*A (*p* < 0.001), respectively. The inhibitory rates for the treatments were 0%, 31.55%, 48.11%, 57.75%, 62.01%, 65.51%, and 73.19%, respectively (Figures 4(a) and 4(c)). In this figure, lines (1) to (7) represent the concentration of extract being 0, 36, 72, 108, 144, 180, and 216 *μ*g·mL^−1^ in the presence of 28 *μ*g·mL^−1^ xanthine, respectively. Hence, it is shown that the electron transfer signal blocking activities (ETSBA) of XOD were modulated by the extract of* Plantaginis Semen*.

The above-mentioned ETSBA characteristics are very valuable. Firstly, it is very easy to eliminate false results as ETSBA not only showed the increased current of the electrochemical constituents, but also displayed the blocked electron transfer signal of XOD. Secondly, an established condition for the evaluation of an extract could be applied for the evaluation of all compounds from the extract. Lastly, the extract could be tested directly and did not need complicated sample pretreatment procedures using a cheap electrochemical station. Hence, ETSBA is very convenient to accurately evaluate the XOD inhibition effect of* Plantaginis Semen *extract.

The IC20 value of the extract has been calculated to be 24.28 *μ*g·mL^−1^. The IC50 value of the extract has been calculated to be 89.14 *μ*g·mL^−1^, which is close to Kong's observation [5]. These results are also similar to another study [16], which may further prove that the electrochemical method could be applied to evaluate XOD inhibitory effects of natural compounds with high sensitivity.

#### 3.4.2. Evaluation of XOD Inhibitory Effect of Compounds with or without Electrochemical Activity via Electron Transfer Signal Blocking Activities

**Figure 5:**
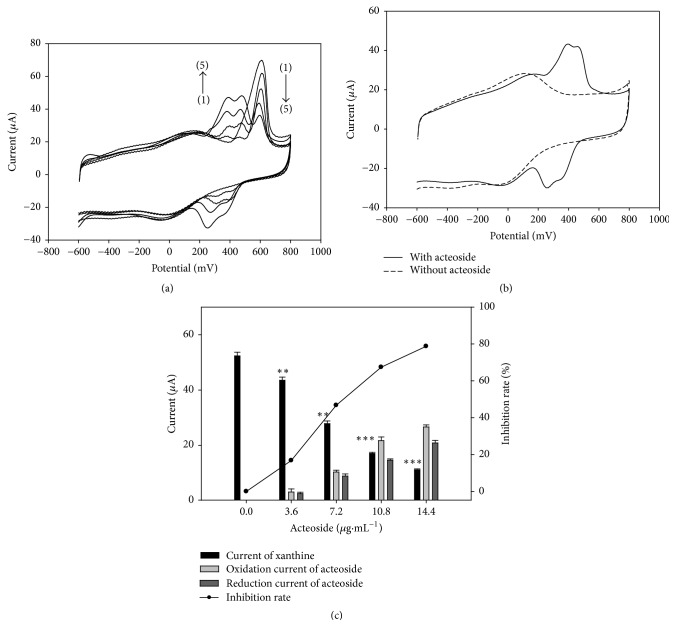
Effects of the representative electron transfer signal blocking activities of acteoside on XOD/DCNTs/GCE. (a) In the presence of xanthine with different concentrations of acteoside. Line (1) to line (5) are XOD/DCNTs/GCE in the presence of 28 *μ*g·mL^−1^ xanthine and 0, 3.6, 7.2, 10.8, and 14.4 *μ*g·mL^−1^ of acteoside, respectively. (b) In the absence and in the presence of acteoside. (c) Relationship between the inhibitory rate and acteoside concentration. ^*∗∗*^*p* < 0.01 and ^*∗∗∗*^*p* < 0.001, compared with control.

The representative natural compounds with XOD inhibitory effects could be divided into two subgroups according to their electrochemical activity. Acteoside is the main active constituent in* Plantaginis Semen*. It was reported that acteoside has XOD inhibition activity [22]. In this work, its inhibition effect was evaluated by ETSBA and the results are shown in Figure 5. It can be seen from Figures 5(a) and 5(b) that the two couples of redox peaks are located at about 200–400 mV. It is obvious that the peaks were caused by the redox of acteoside. As shown in Figure 5, the peak current increased with the successive addition of 0, 3.6, 7.2, 10.8, and 14.4 *μ*g·mL^−1^ acteoside (for line (1) to line (5), respectively), while the peak current located at about 610 mV decreased accordingly (Figures 5(a) and 5(c)). The corresponding current was 52.36 ± 1.35 *μ*A (*p* < 0.01), 43.53 ± 1.15 *μ*A (*p* < 0.01), 27.87 ± 0.94 *μ*A (*p* < 0.001), 17.06 ± 0.43 *μ*A (*p* < 0.001), and 11.13 ± 0.22 *μ*A (*p* < 0.001). The inhibition rates were 0%, 16.86%, 46.88%, 67.42%, and 78.30%, respectively. The relationships of the response current of ETSBA and inhibition rate of acteoside are summarized in Figure 5(c). The IC20 value of the acteoside has been calculated to be 3.88 *μ*g·mL^−1^. The IC50 value of acteoside was calculated to be 7.55 *μ*g·mL^−1^. This value is also smaller than the 12.5 *μ*g·mL^−1^ that was reported in previously published data [22], which may be due to the higher sensitivity of ETSBA [16]. Obviously, the results proved that the natural compound group with electrochemical activity and high XOD inhibitory effect could be accurately evaluated by ETSBA. Furthermore, similar to that of the extract, it not only can show the increased current of the acteoside, but also can display the blocked electron transfer signal of XOD, which helps to eliminate false results.

**Figure 6:**
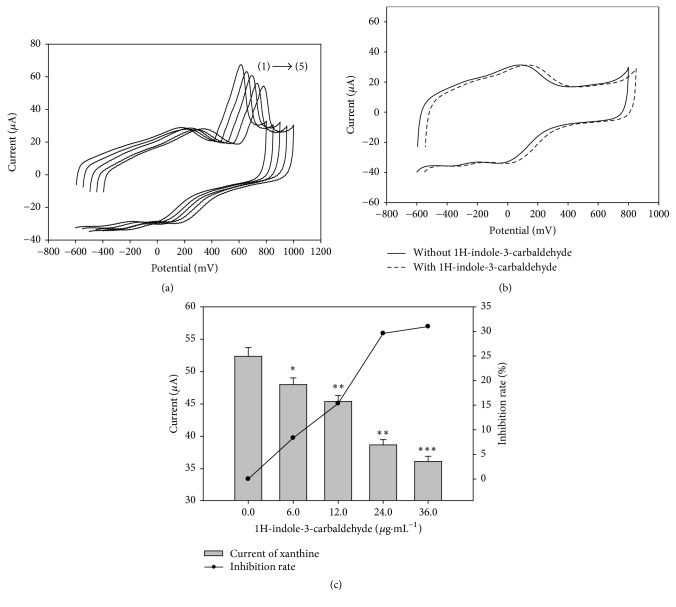
Effects of the representative electron transfer signal blocking activities of 1H-indole-3-carbaldehyde on XOD/DCNTs/GCE. (a) In the presence of xanthine with different concentrations of 1H-indole-3-carbaldehyde. The lines (1) to (5) are XOD/DCNTs/GCE in the presence of 28 *μ*g·mL^−1^ xanthine and 0, 6.0, 12.0, 24.0, and 36.0 *μ*g·mL^−1^ of 1H-indole-3-carbaldehyde, respectively. (b) In the absence and in the presence of 1H-indole-3-carbaldehyde. (c) Relationship between the inhibitory rate and 1H-indole-3-carbaldehyde concentration. ^*∗*^*p* < 0.05, ^*∗∗*^*p* < 0.01, and ^*∗∗∗*^*p* < 0.001, compared with control.

1H-indole-3-carbaldehyde has never been reported with XOD inhibition activity before. Its electrochemical characteristics and the inhibitory effect on XOD were studied by ETSBA and the results were listed in Figure 6 (the CVs were shifted to left in sequence by every 50 mV). It can be seen from Figures 6(a) and 6(b) that 1H-indole-3-carbaldehyde did not show any electrochemical activity. While the concentration of 1H-indole-3-carbaldehyde increased (from line (1) to line (5) in Figure 6(a) is 0, 6.0, 12.0, 24.0, and 36.0 *μ*g·mL^−1^, respectively), the currents located at 610 mV decreased accordingly (Figures 6(a) and 6(c)). The corresponding current was 52.36 ± 1.35 *μ*A, 47.99 ± 1.03 *μ*A (*p* < 0.05), 45.39 ± 0.91 *μ*A (*p* < 0.01), 38.68 ± 0.82 *μ*A (*p* < 0.01), and 36.13 ± 0.79 *μ*A (*p* < 0.001), respectively. The inhibition rate for the compound was 0%, 8.35%, 15.36%, 29.60%, and 30.99%, respectively. The inhibitory effect of 1H-indole-3-carbaldehyde could not exceed 50%. Hence the IC20 value was tested and calculated to be 16.16 *μ*g·mL^−1^, which shows that it is a low activity XOD inhibitor. As the peak current located at about 610 mV came exclusively from the electron transfer signal of the enzyme catalytic reaction, the XOD inhibitory effects of compound group without any electrochemical activity could be evaluated by ETSBA and caused no false results even if it is a low XOD inhibitor.

#### 3.4.3. Evaluation of Compounds without XOD Inhibitory Effect While with or without Electrochemical Activity via Electron Transfer Signal Blocking Activities

**Figure 7:**
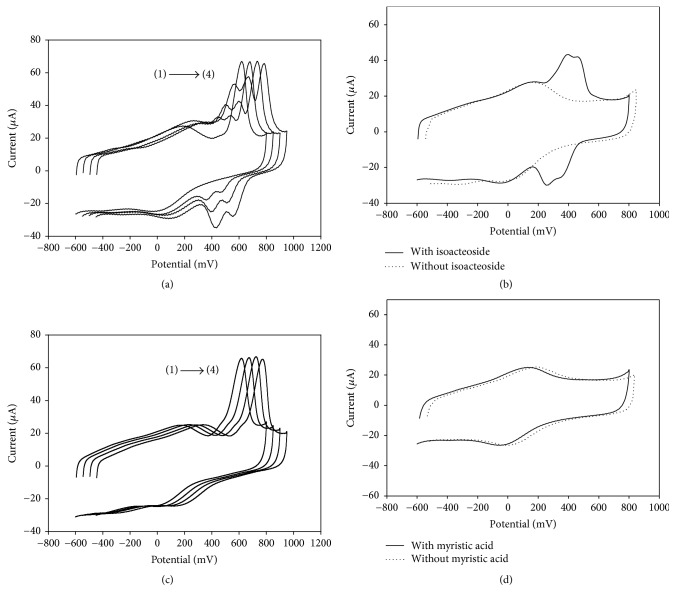
Effects of the representative electron transfer signal blocking activities of isoacteoside and myristic acid on XOD/DCNTs/GCE. (a) In the presence of xanthine with different concentration of isoacteoside. The lines (1) to (4) are XOD/DCNTs/GCE in the presence of 28 *μ*g·mL^−1^ xanthine and 0, 3.6, 7.2, and 10.8 *μ*g·mL^−1^ of isoacteoside, respectively. (b) In the absence and in the presence of different concentrations of isoacteoside. (c) In the presence of xanthine and different concentrations of myristic acid. The lines (1) to (4) are XOD/DCNTs/GCE in the presence of 28 *μ*g·mL^−1^ xanthine and 0, 3.0, 12.0, and 30.0 *μ*g·mL^−1^ myristic acid, respectively. (d) In the absence and presence of myristic acid.

Isoacteoside is an isomer of acteoside. It can be seen from Figures 7(a) and 7(b) that isoacteoside is an electrochemical active compound. The peak current located at about 200–500 mV increased with the increased concentration of 0, 3.6, 7.2, and 10.8 *μ*g·mL^−1^ of isoacteoside (for lines (1) to (4), respectively), which is similar to that of acteoside. This phenomenon is reasonable as the electrochemical active functional group of isoacteoside is the same as that of acteoside. The currents located at 610 mV remains constant, and there was no significant difference observed for isoacteoside. This means that isoacteoside could not affect the electron transfer signal blocking activities and it is not a XOD inhibitor. This result is very trusted as the oxidation-reduction current of isoacteoside increased with the increased concentration, while the current of electron transfer signal was kept constant with the successive addition of isoacteoside. The result is also consistent with previously published work [23]. It is reported that acteoside could enter into the active site of XOD and form hydrogen bonding with amino acid residues (such as Lys-1045, Arg-880, Arg-912, Glu-1261, and Gln-1194) [24] to exhibit XOD inhibitory effects. For isoacteoside, the reason why no XOD inhibition effect was seen could be ascribed to its stereo structure. As shown in Figure 2, the caffeoyl- of acteoside is located at C-4 of glucose, while caffeoyl- of isoacteoside is embedded at the C-6 of glucose. The difference in structure of isoacteoside may lead to a different stereo structure, which may obstruct isoacteoside entering XOD center and format hydrogen. Hence, the results showed that the compounds group without XOD inhibitory effect with electrochemical activity could be accurately evaluated by ETSBA. Furthermore, the results also proved that ETSBA could accurately evaluate the XOD inhibitory effect of isomers.

Myristic acid is a long chain fatty acid and its inhibitory effect on XOD is shown in Figures 7(c) and 7(d) (the concentration in Figure 7(c) of myristic acid from lines (1) to (4) is 0, 3.0, 12.0, and 30.0 *μ*g·mL^−1^, respectively). To clearly illustrate the inhibition effects of isoacteoside and myristic acid on XOD, the CVs were successively shifted to the left by every 50 mV, respectively. As seen from Figures 7(c) and 7(d), there is no CVs peak except xanthine, which is located at about 610 mV, meaning that myristic acid is not an electrochemical active compound. The currents located at 610 mV also stayed constant, and no significant difference was observed for myristic acid. This indicated that this compound could not affect electron transfer signal blocking activities and not be an XOD inhibitor. Hence, the results showed that the compounds groups without XOD inhibitory effect and electrochemical activity could be easily evaluated by ETSBA.

## 4. Conclusions

The mechanism of electron transfer blocking activities (ETSBA) is completely different from other traditional methods. The XOD inhibitory effects of the extract and four representative compounds from Plantaginis Semen can be evaluated by ETSBA as a simple and sensitive and technique. Plantaginis Semen possesses significant XOD inhibitory effects and can be applied for treatment of gout and hyperuricemia as it contains XOD inhibitors. The IC50 value of the extract can be accurately detected, while the activity of acteoside is significantly higher. The IC50 values for the other three single compounds cannot be quantified. Thus, this ETSBA technique can be used to accurately measure XOD inhibitory effects using the electrochemical activity assessment. It appears that the ETSBA technique may be a novel way to effectively evaluate XOD inhibitory activities.

## Figures and Tables

**Table 1:** The electrochemical activity and XOD inhibitory activity of Plantaginis Semen extract and four representative compounds.

	Name	Electrochemical activity	XOD inhibitory activity	IC/*μ*g·mL^−1^	
				IC20	IC50
Extract	Plantaginis Semen	Yes	High	24.28	89.14
Compound 1	Acteoside	Yes	High	3.88	7.55
Compound 2	1H-indole-3-carbaldehyde	No	Low	16.16	- -
Compound 3	Isoacteoside	Yes	No	- -	- -
Compound 4	Myristic acid	No	No	- -	- -
